# Supplementary material for: TC10 differently controls the dynamics of Exo70 in growth cones of cortical and hippocampal neurons
Source: Biophys Rep (N Y). 2024 Nov 7;4(4):100186. doi: 10.1016/j.bpr.2024.100186 (PMC11617994; doi:10.1016/j.bpr.2024.100186)
Supplement: Document S1. Figures S1–S5, Tables S1–S3, and supporting methods [file mmc1.pdf]

Biophysical Reports, Volume *4*

Supplemental information

TC10 differently controls the dynamics of Exo70 in growth cones of cortical and hippocampal neurons

Hiteshika Gosain and Karin B. Busch

Supporting Material

TC10 differently controls the dynamics of Exo70 in growth cones of cortical and hippocampal neurons

Running title: Exo70 dynamics controlled by TC10

Hiteshika Gosain[1], Karin B. Busch[1]*

[1]*Institute of Integrative Cell Biology and Physiology, Department of Biology, University of Muenster, 48149 Münster, North-Rhine-Westphalia, Germany*

*Corresponding author

Karin Busch, e-mail: buschkar@uni-muenster.de

Supplementary Methods

Co-Localization of Exo70 and actin

Hippocampal neurons were a gift from A. Püschel. Exo70 was immune-stained with anti-Exo70 (rb) (Proteintech (12014-1-AP) using a dilution of 1:50, the 2nd antibody was anti-rb-Alexa555 (Invitrogen A21147) used in a dilution of 1:500. Actin was stained with Alexa488-Phalloidin (1 µM, Invitrogen A12379). Exo70/Alexa555 was excited with 551 nm, Alexa488-Phalloidin with 488 nm. Imaging was performed with an Elyra7-TIRF microscope. Two aligned Hamamatsu ORCA-Fusion BT sCMOS cameras were used to image fluorescent Exo70 and actin.

Supplementary Figures

Figure S 1: Colocalization of Exo70 and TC10 in HeLa cells. (A) Endogenous TC10 (green) and (b) endogenous Exo70 (red), immune-staining. (c) Merge of Exo70 and TC10. TC10 and Exo70 are found both at the plasma membrane and as puncta. (d, e, f) Zoomed in regions of figures (a, b, c) (white boxes), size 100x100 pixels. Colocalization of Exo70 and TC10 are shown as yellow spots in (c) and (f). Primary/secondary antibody are Exo70/AF555, TC10/AF488. (g) The threshold for both the green (TC10) and red (Exo70) channels was adjusted, and colocalization was performed in Imaris™ (Bitplane) in an area of 100x100 pixels. Colocalization is indicated by white spots. (h) Scatter plot to determine the Pearson's coefficient. (i) Calculated colocalization (%) averaged over 30 images (100x100 px). Scale bars: 10 µm (a,b,c), 1 µm (d,e,f).

Figure S 2: **Effect of TC10 on Exo70 dynamics in the soma of cortical neurons**. (A) Single particle imaging of Exo70 in the absence and presence of TC10 and TC10 variants in the soma, a single frame is shown. (B) Cumulative image of 5000 frames with localized Exo70 particles. (C) Trajectory map of Exo70 in the absence and presence of TC10 and TC10 variants. Each trajectory is labelled in a colour related to the mean diffusion coefficient. Cumulative image of 5000 frames recoded with 58.8 Hz. (D) Characterization of Exo70 diffusion in the soma: mean square displacements (MSD) over time. (E) Change of the diffusion coefficient *D* over time (-log D). (F) Diffusion coefficients as -log *D*. The box limit indicates the 25th and 75th percentiles; whiskers extend to 1.5 times the interquartile range of the 25th and 75th percentiles; a straight line in the box plot gives the median values and this value is

presented next to the box; the significant p values were estimated using the Kruskal-Wallis ANOVA test followed by post-hoc analysis. N=2.

Figure S 3: Confinement and anomalous diffusion of Exo70 at the plasma membrane in HeLa cells and cortical neurons. (A) Means square displacements. (B) Dissection of motion behavior. The pie chart shows the respective population of Exo70 following anomalous or confined motion (grey part, $\alpha<1$), Brownian motion (red part, $\alpha=1$) and directed motion (green portion, $\alpha>1$).

Figure S 4: **Partial co-localization of Exo70 and actin in hippocampal neurites.** (A) Immunolabeled endogenous Exo70 and FITC-Phalloidin stained actin show co-localization in hippocampal neurons. (B) Several regions of interest (rois) were analyzed for colocalization using the Colocalization ImageJ plugin. The Pearson's colocalization factor was calculated (graph). Each dot represents data from a single roi.

Figure S 5: Exo70 mobility in HeLa cells is influenced by actin. Destruction of the actin skeleton in HeLa cells by latrunculin and its effect on Exo70-HaloTag mobility shown as means square displacement (MSD) over time. Framed area indicates short-term movement.

Figure S 1

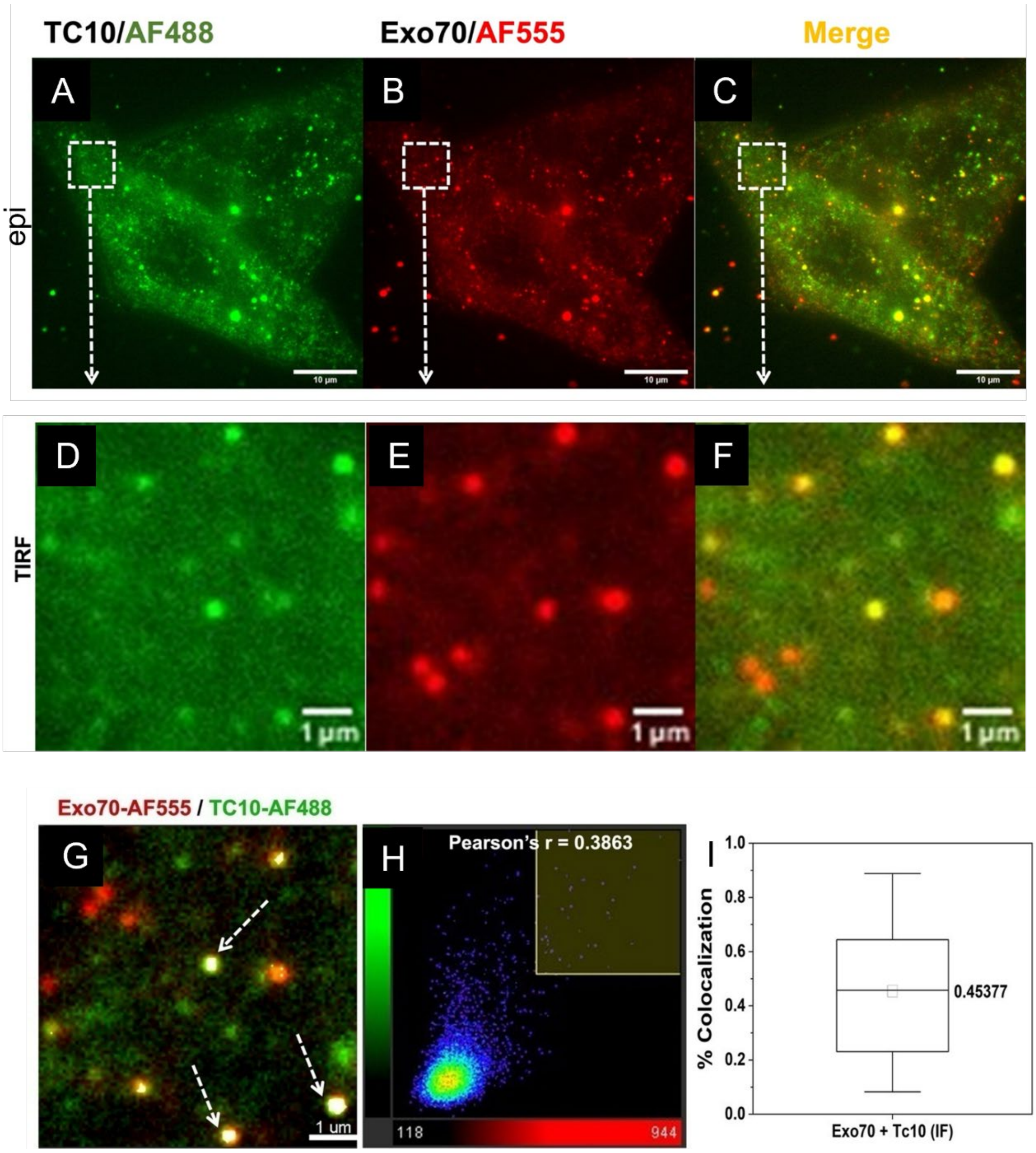
TC10/AF488
Exo70/AF555
Merge
A
B
C
epi
10 µm
D
E
F
TIRF
1 µm
Exo70-AF555 / TC10-AF488
G
1 um
H
Pearson's r = 0.3863
118
944
I
% Colocalization
1.0
0.8
0.6
0.4
0.2
0.0
0.45377
Exo70 + Tc10 (IF)

Figure S 2

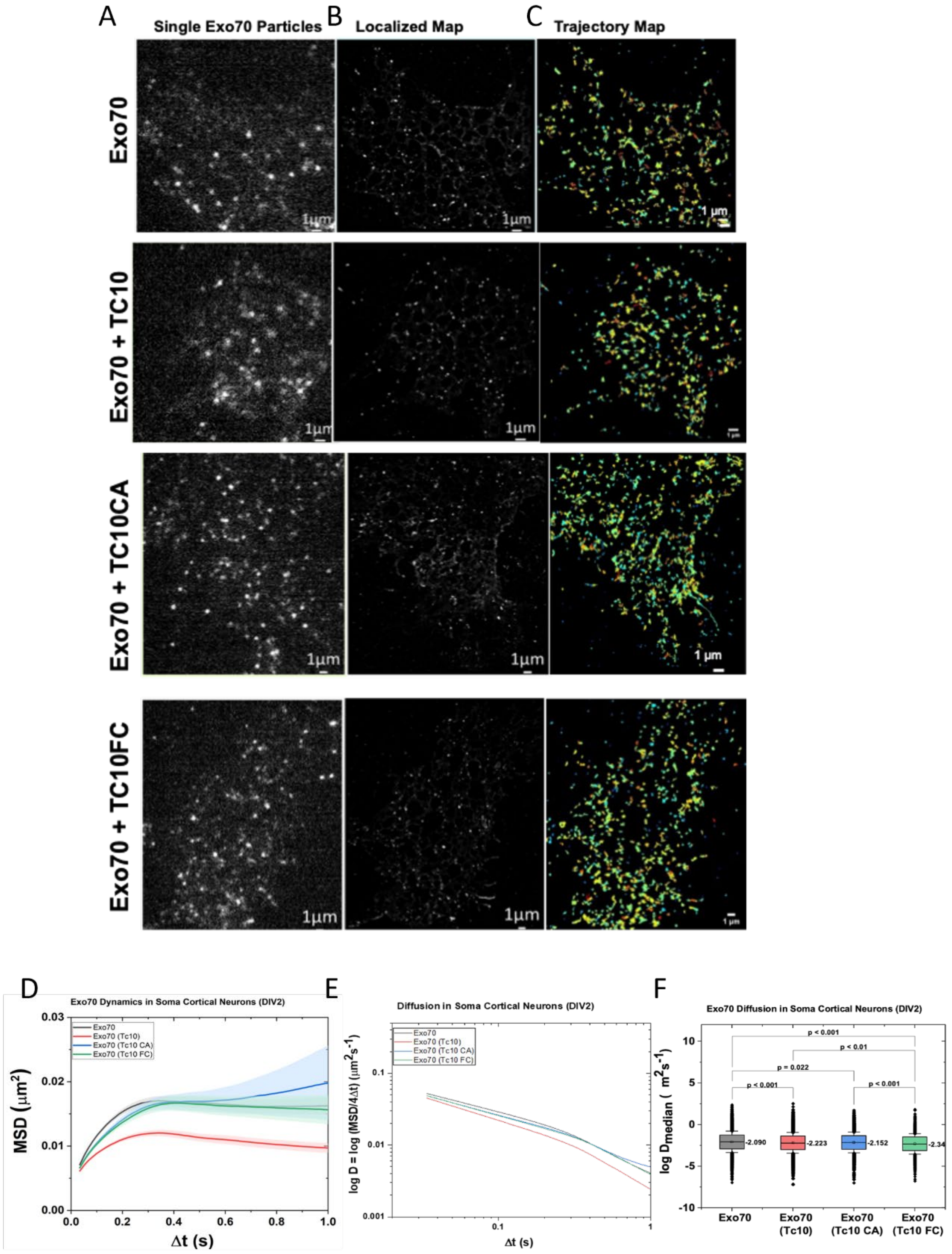
A
Single Exo70 Particles
B
Localized Map
C
Trajectory Map
Exo70
Exo70 + TC10
Exo70 + TC10CA
Exo70 + TC10FC
1µm
1 µm
D
Exo70 Dynamics in Soma Cortical Neurons (DIV2)
Exo70
Exo70 (Tc10)
Exo70 (Tc10 CA)
Exo70 (Tc10 FC)
MSD (µm²)
Δt (s)
E
Diffusion in Soma Cortical Neurons (DIV2)
log D = log (MSD/4Δt) (µm²s⁻¹)
Δt (s)
F
Exo70 Diffusion in Soma Cortical Neurons (DIV2)
p < 0.001
p < 0.01
p = 0.022
p < 0.001
p < 0.001
-2.090
-2.223
-2.152
-2.34
log D median (m²s⁻¹)
Exo70
Exo70 (Tc10)
Exo70 (Tc10 CA)
Exo70 (Tc10 FC)

Figure S 3

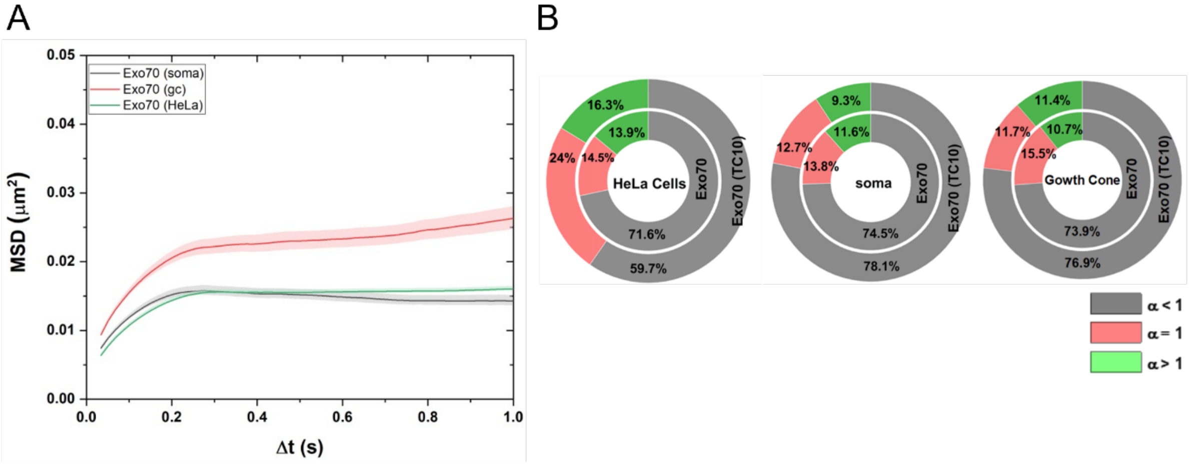
A
B
Exo70 (soma)
Exo70 (gc)
Exo70 (HeLa)
MSD (μm²)
Δt (s)
HeLa Cells
soma
Gowth Cone
Exo70
Exo70 (TC10)
16.3%
13.9%
24%
14.5%
71.6%
59.7%
9.3%
11.6%
12.7%
13.8%
74.5%
78.1%
11.4%
10.7%
11.7%
15.5%
73.9%
76.9%
α < 1
α = 1
α > 1

Figure S 4

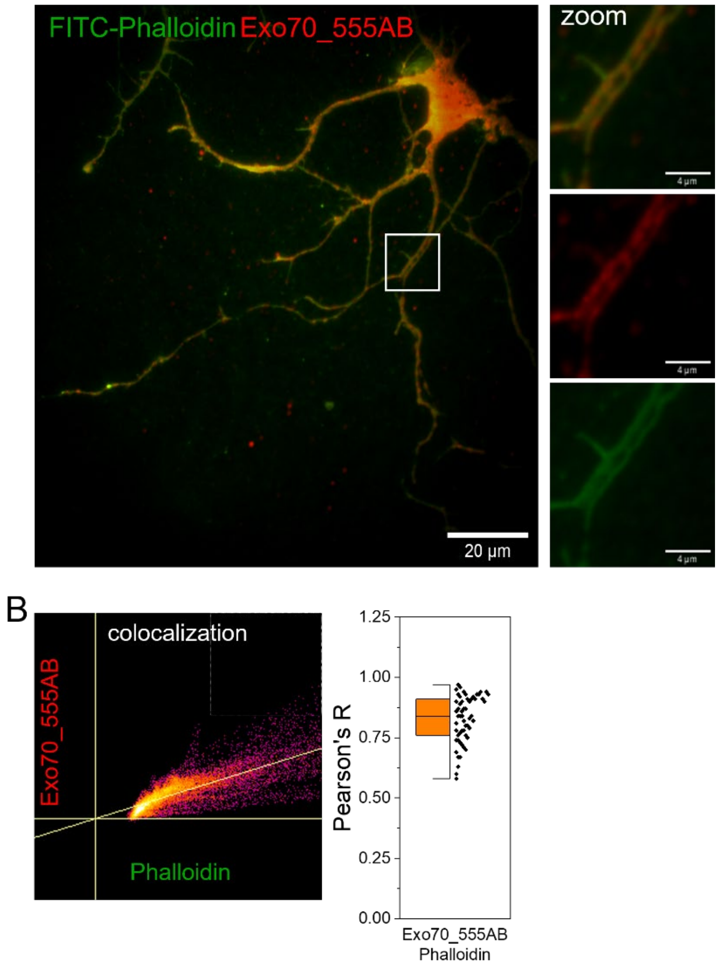
FITC-Phalloidin Exo70_555AB
zoom
20 µm
4 µm
4 µm
4 µm
B
colocalization
Exo70_555AB
Phalloidin
Pearson's R
1.25
1.00
0.75
0.50
0.25
0.00
Exo70_555AB
Phalloidin

Figure S 5

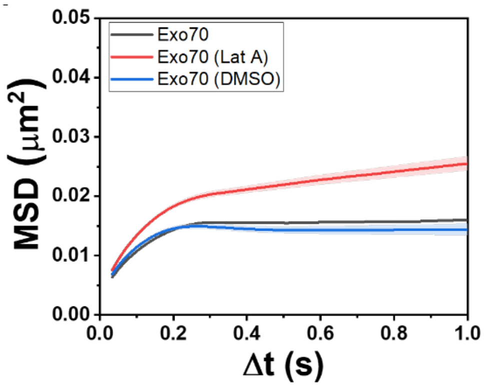

0.05
0.04
0.03
0.02
0.01
0.00
MSD (μm²)
0.0
0.2
0.4
0.6
0.8
1.0
Δt (s)
Exo70
Exo70 (Lat A)
Exo70 (DMSO)

Supplementary Tables

Table 1: Effect of TC10 on the diffusion of Exo70 in nonpolar HeLa cells, N=3.

HeLa cells	Total # tracks	$\mathcal{D}_{median}$±s.e.m. (µm²s⁻¹)	α_{mean} ± s.e.m.	Number of cells
Exo70	22724	0.011 ± 0.001	0.776 ± 0.003	20
Exo70 (TC10)	11644	0.031 ± 0.002	0.832 ± 0.005	20
Exo70 (TC10CA)	9249	0.026 ± 0.003	0.830 ± 0.005	20
Exo70 (TC10FC)	16188	0.021 ± 0.002	0.811 ± 0.004	20

Table 2: Effect of TC10 on the diffusion of Exo70 in the axonal growth cone of cortical neurons, N=2.

Growth Cone Cortical Neurons (DIV2)	Total tracks	$\mathcal{D}_{median}$±s.e.m. ($\mu m^2 s^{-1}$)	α_{mean} ± s.e.m.	Number of cells
**Exo70**	6938	0.011 ± 0.002	0.749 ± 0.006	13
**Exo70 (TC10)**	9108	0.007 ± 0.003	0.731 ± 0.006	13
**Exo70 (TC10CA)**	4402	0.007 ± 0.005	0.745 ± 0.007	12
**Exo70 (TC10FC)**	4949	0.006 ± 0.003	0.724 ± 0.007	12

Table 3: Effect of TC10 on the diffusion of Exo70 in the axonal growth cone of hippocampal neurons. N=2.

Growth Cone Hippocampal Neurons (DIV3)	Total tracks	$\mathcal{D}_{median}$±s.e.m. ($\mu m^2 s^{-1}$)	α_{mean} ± s.e.m.	Number of cells
**Exo70**	5544	0.015 ± 0.001	0.778 ± 0.007	9
**Exo70 (TC10)**	3407	0.017 ± 0.002	0.770 ± 0.009	8
**Exo70 (TC10CA)**	5229	0.016 ± 0.003	0.794 ± 0.008	8
**Exo70 (TC10FC)**	4552	0.016 ± 0.003	0.771 ± 0.008	8
