# Supplementary material for: TC10 differently controls the dynamics of Exo70 in growth cones of cortical and hippocampal neurons
Source: Biophys Rep (N Y). 2024 Nov 7;4(4):100186. doi: 10.1016/j.bpr.2024.100186 (PMC11617994; doi:10.1016/j.bpr.2024.100186)
Supplement: Document S2. Article plus supporting material [file mmc2.pdf]

Biophysical Reports

REPORT

TC10 differently controls the dynamics of Exo70 in growth cones of cortical and hippocampal neurons

Hiteshika Gosain[1] and Karin B. Busch[1,*]
[1]Institute of Integrative Cell Biology and Physiology, Department of Biology, University of Muenster, Münster, North-Rhine-Westphalia, Germany

ABSTRACT The exocyst is an octameric protein complex that acts as a tether for GOLGI-derived vesicles at the plasma membrane during exocytosis. It is involved in membrane expansion during axonal outgrowth. Exo70 is a major subunit of the exocyst complex and is controlled by TC10, a Rho family GTPase. How TC10 affects the dynamics of Exo70 at the plasma membrane is not well understood. There is also evidence that TC10 controls Exo70 dynamics differently in nonpolar cells and axons. To address this, we used super-resolution microscopy to study the spatially resolved effects of TC10 on Exo70 dynamics in HeLa cells and the growth cone of cortical and hippocampal neurons. We generated single-particle localization and trajectory maps and extracted mean square displacements, diffusion coefficients, and alpha coefficients to characterize Exo70 diffusion. We found that the diffusivity of Exo70 was different in nonpolar cells and the growth cone of neurons. TC10 stimulated the mobility of Exo70 in HeLa cells but decreased the diffusion of Exo70 in the growth cone of cortical neurons. In contrast to cortical neurons, TC10 overexpression did not affect the mobility of Exo70 in the axonal growth cone of hippocampal neurons. These data suggest that mainly exocyst tethering in cortical neurons was under the control of TC10.

WHY IT MATTERS Exo70 is a central subunit of the vesicle tethering complex in exocytosis. Exocytosis is important, for example, in the regeneration of nerves. The Rho-GTPase TC10 is a regulator of exocytosis, but how TC10 influences Exo70 is not fully understood. Here, we used single-molecule tracking to quantify the effect of TC10 on the dynamics of Exo70 at the plasma membrane. We showed that TC10 slowed Exo70 dynamics in the axonal growth cone of cortical neurons consistent with stimulated vesicle binding but found no obvious effect of TC10 on Exo70 dynamics in hippocampal neurons. Moreover, the effect of TC10 on Exo70 was again different in nonpolar HeLa cells, suggesting that the control of Exo70 by TC10 is a complex process.

INTRODUCTION

Exocytosis is a dynamic process contributing to cellular autophagy, polarization, membrane expansion, and vesicle fusion. During early neuronal differentiation, neurite outgrowth and extension are key events (1,2) that require membrane surface expansion by exocytosis at the leading edge (3). The tethering of exocytic vesicles is mediated by the exocyst complex, which is composed of two subcomplexes: the Exo70 subcomplex with Sec10, Sec15, and Exo84 and a subcomplex composed of Sec3, Sec5, Sec6, and Sec8 (4). Exo70 is the determinant for the assembly of the whole complex and thus determines exocytosis. The dynamics of Exo70 at the plasma membrane (PM) is mediated by TC10, a member of the Rho family of GTPase proteins (5). GTP hydrolysis of TC10 promotes neurite outgrowth by promoting exocytosis (6,7). GTP hydrolysis of TC10 promotes the tethering of vesicles and, subsequently, the release of Exo70 from the PM. It is not clear whether the effect of TC10 on Exo70 dynamics is quantitively the same in all neurons, as other Rho GTPase also modulate axon formation (8). To address this, we compared the effect of TC10 on Exo70 dynamics in cortical and hippocampal neurons. We analyzed the dynamics of Exo70 at the PM in in early-stage differentiating neurons using super-resolution imaging and quantitative analysis. We quantified the mobility of Exo70 in the presence of TC10 and mutants of TC10 by single-particle tracking (SPT) using total internal reflection fluorescence (TIRF) microscopy. The constitutively active TC10/Q67L (or TC10CA) and the fast-cycling TC10/34L (TC10FC) were used to study the influence of mutants

Submitted June 3, 2024, and accepted for publication November 5, 2024.
*Correspondence: buschkar@uni-muenster.de
Editor: Ulrike Endesfelder.
https://doi.org/10.1016/j.bpr.2024.100186
© 2024 The Author(s). Published by Elsevier Inc. on behalf of Biophysical Society.
This is an open access article under the CC BY license (http://creativecommons.org/licenses/by/4.0/).

TABLE 1 General material

Materials	Supplier	Catalog number
Alexa Fluor 647 goat anti-mouse IgG (H+L)	Thermo Fischer Scientific	A21235
B27 supplement 50×	Thermo Fischer Scientific	17504044
Cell counter	Marienfeld Superior	0.0025 mm^2
Dulbecco's modified Eagle's medium-high glucose (DMEM)	Sigma-Aldrich	D6546
Dulbecco's phosphate-buffered saline	Sigma-Aldrich	D8537
Fetal bovine serum	PAN Biotech	P30-3031
Gentamicin	Thermo Fischer Scientific	15750060
HEPES solution	Sigma-Aldrich	H0887
Janelia Fluor 646 Halo Tag	Promega Corporation	GA1120
Janelia Fluor 549 SNAP Tag	Tocris Biotechne	6147
L-glutamine solution	Sigma-Aldrich	G7513
Lipofectamine 3000 Reagent Transfection Kit	Thermo Fischer Scientific	L3000-001
Nonessential amino acid solutions	PAN Biotech	P08-32100
Neurobasal medium (1×)	Gibco	12348–017
Paraformaldehyde	BioChemica	A3813,0250
Penicillin-streptomycin	PAN Biotech	P06-07100
Sodium pyruvate solution	Sigma-Aldrich	S8636
TC-Flasche T25, Stand., Bel. Ka.	SARSTEDT AG & Co. KG	83.3910.002
μ-Dish 35 mm high glass bottom	Ibidi GmbH	81158

on Exo70 dynamics. Our study revealed that TC10-mediated control of Exo70 dynamics is distinct between hippocampal and cortical neurons.

MATERIALS AND METHODS

Cell culture and cultivation

HeLa cells (purchased from DSMZ, #ACC 57) were chosen as a nonpolar model cell line. HeLa cells were cultured in Dulbecco's modified Eagle medium (DMEM), 10% fetal bovine serum, 1% nonessential amino acids, 1% HEPES buffer solution, 1% L-glutamine, and 1% penicillin as an antibiotic. Table 1 lists the general material used and Table 2 the culture medium compositions.

Primary rat embryo neurons were obtained from a rat hippocampus on embryonic day 18 (E18). All animal protocols were performed in accordance with the guidelines of the North Rhine-Westphalia State Environment Agency (LANUV). Rats were maintained at the animal facility of the Institute for Integrative Cell Biology and Physiology (University of Münster) under standard housing conditions with a 12 h light/dark cycle at constant temperature (23°C) and with ad libitum supply of food and water. Timed pregnant rats were set up in house. Pregnant rats were anesthetized by exposure to isoflurane, followed by decapitation and primary cultures prepared from embryos at E18. During dissection, neurons from all embryos (regardless of sex) were pooled.

Neurons were grown in supplemented neurobasal medium (NBM++) and kept at 37°C and 5% CO_2. To obtain polar neurons, hippocampi were isolated from rat embryos (at E18) and collected in HBSS on ice. Hippocampi were incubated at 37°C for 8 min in 2 mL trypsin (not resuspended). They were washed five times in prewarmed DMEM++ before being resuspended in 2 mL DMEM++ with a 1 mL pipette tip after aspirating trypsin. Neurons were then seeded on poly-ornithine-coated dishes with NBM++ medium and incubated at 37°C with 5% CO_2.

For microscopy, 100,000 neurons were seeded on poly-ornithine-coated 3 cm glass-bottom dishes. Neurons were cultured in DMEM++ medium, which was replaced with conditioned media after 3 h and placed in an incubator at 37°C and 5% CO_2. The day when neurons were seeded is termed "day *in vitro* 0" (DIV0). The first day following seeding is known as DIV1, and the days after that are known as DIV2, DIV3, and so on.

Transfection

HeLa cells were transfected with Lipofectamine 3000 according to the manufacturer's instructions and neurons by the calcium phosphate transfection procedure. After washing, neurons were supplied with precollected conditioned medium and left for 3 days to mature. At DIV3, live neurons were imaged.

Staining self-labeling tags for live-cell imaging

The protein of interest was genetically fused to either the HaloTag (Halo) or the SNAPtag (SNAP) at the C-terminus of the Exo70 protein sequence resulting in Exo70-Halo and the N-terminus of TC10 (SNAP-TC10). For HaloTag labeling, we used a fluorescent derivative of 1-chlorohexane as a substrate, the HaloTag ligand (HTL), which forms a covalent bond with the HaloTag. For SNAPtag labeling, a fluorescent benzyl-guanine (BG) was used. Janelia Fluor (JF) dyes JF646-HTL (500 pM) and JF549-BG (2 nM) were used in single molecular studies performed with the TIRF microscope. Cells were incubated with the dye-substrates for 30 min at 37°C and 5% CO_2 and washed and imaged in a prewarmed imaging medium. For confocal imaging, 50 nM JF646-HTL and 50 nM JF549-BG were used.

Immunostaining

Cells were fixed with 4% paraformaldehyde containing 15% sucrose for 10 min at room temperature. Samples were washed three times in PBS, then treated with 50 mM ammonium chloride (NH_4Cl) to eliminate free aldehyde groups. Cells were permeabilized with 0.1% Triton X-100 in PBS for 10 min. After washing with PBS, blocking was done with 2% goat serum and 3% bovine serum albumin (1 h at room temperature in a dark, humid environment). Residual blocking buffer was aspirated after 1 h. The primary antibody against endogenous Exo70 (12014-1-AP, Proteintech) was used at a dilution of 1:50 (Table 3). The secondary antibody conjugated with Alexa Fluor 555 or Alex Fluor 488 was used at a dilution of 1:500.

TABLE 2 Cell culture medium compositions

Medium	Composition
DMEM++ (growth medium)	1× DMEM with phenol red, 10% heat-inactivated FBS, 2 mM L-glutamine, NEAA (1%), HEPES (1 μM), and 50 μg/mL penicillin-streptomycin
DMEM+++ (imaging medium)	1× DMEM without phenol red, 10% heat-inactivated FBS, 2 mM L-glutamine, NEAA (1%), HEPES (1 μM), and 50 μg/mL penicillin-streptomycin
NBM++ (conditioned medium)	50 mL neurobasal medium (NBM), 1 mL B27, 500 μL 100× glutamine, and 125 μL gentamicin

Cloning of Exo70-Halo

Exo70 was amplified from pEGFP-C3-Exo70 (Addgene plasmid #53761) using the primers 5′-CGGAATTCTG ATGATTCCCC CACAG GAGG-3′ and 5′-GCGTCGACAC GGCAGAGGTG TCGAAAAGGC-3′ and inserted into the pSEMS-HaloTag vector.

Microscopy

For confocal imaging, an inverted cLSM (Leica TCS SP8 FLIM system) was used. The microscope was equipped with a unique acoustic optical deflector, two photomultiplier tubes, two highly sensitive hybrid GAsP (HyD) detectors, a 63× water objective (numerical aperture = 1.2), and a pulsed white light laser. Measurements were done at 37°C and 5% CO_2. An image format of 1024 × 1024 pixels was used with a scan speed of 700 Hz to obtain images with a high signal/noise ratio. z stacks were acquired and processed in FIJI to create an averaged intensity projection of all the images in the stack.

TIRF microscopy

An inverted TIRF microscope (Xplore, Olympus) was used for SPT studies. The microscope is equipped with a TIRF objective (100× oil, numerical aperture = 1.49), an Optosplit IV (Cairn) for simultaneous recording of four channels, and four diode lasers (wavelengths of 405, 488, 560, and 640 nm) connected by an optical fiber to four independently controlled TIRF modules. To reduce wide-field illumination, a collimated beam of light was generated by focusing into the back focal plane of the objective. The output intensities of the diode laser in the system were controlled on a microsecond timescale by an acoustical optical tunable filer. To image single molecules, we used a highly sensitive sCMOS camera (ORCA Fusion-BT, Hamamatsu) at a pixel size of 65 nm. 5000 frames with a constant time interval (58.8 Hz) were usually recorded. The image format for a single channel was set to 500 × 500 pixels.

SPT and data processing

For tracking analysis, the raw images were processed in FIJI using the Trackmate plugin to extract track information for the analysis (9). This program takes the simplest approach possible by detecting each particle's nearest neighbors in a circular region in successive frames. Spots were detected using the "LoG detector" filter. In combination with the median filter and subpixel localization, the threshold was modified according to the signal intensity. The data were visualized using the "HyperStack Displayer" in uniform color. The tracking was done using the simple LAP tracker (linking conditions: maximum distance, gap-closing conditions, maximum distance). The generated tracks were further filtered using a "number of spots in tracks" filter. This filter was used to make sure the tracks were long, to reduce statistical errors and discard tracks produced by blinking occurrences. Resulting tracks (saved as a ".XML" file) were further analyzed in MATLAB using the @msdanalyser to get ensemble mean-square displacement (MSD) curves, diffusion coefficient distributions, and alpha coefficient (α) distributions (10). Generally, ensemble MSD curves were plotted after weighted averaging over all particles. Diffusion coefficients were extracted from the MSD curves using power law and fitted with a linear function. The first 25% (200 ms) of the trajectory was fitted. The threshold for the quality of the fit was R^2 coefficients > 0.8. The fits gave us the values of α and D for individual tracks according to the models described by Qian (11).

TABLE 3 List of antibodies for immunofluorescence staining

Antibody target	Specification	Provider	Ordering number
Exo70	mouse monoclonal	Santa Cruz	sc-365825
TC10	rabbit polyclonal	Proteintech	17805-1-AP
Alexa Fluor 488	goat anti-rabbit	Thermo Fischer Scientific	A-11008
Alexa Fluor 555	goat anti-mouse	Thermo Fischer Scientific	A21147

Statistical analysis

For statistical analysis, Origin software was used for the analysis of α and D. Usually, the Kruskal-Wallis ANOVA test was followed by post hoc analysis.

RESULTS

First, we checked whether Exo70 and TC10 colocalize. Therefore, we immunostained both proteins in HeLa cells. TC10 and Exo70 were found in the PM but also in puncta-like structures in the cytosol. About 45% of Exo70 and TC10 signals colocalized, indicating an interaction (Fig. S1). To test whether TC10 affects Exo70 dynamics, we expressed tagged versions of Exo70 and TC10 in HeLa cells and cortical neurons. This allowed us to conduct single-particle localization and SPT to resolve the effect of TC10 on the spatiotemporal behavior of Exo70 at the PM under TIRF excitation. For live-cell imaging, Exo70 was genetically fused to the self-labeling HaloTag at its C-terminus, annotated as Exo70-Halo. TC10 was genetically fused to the SNAP tag at N-terminus, called SNAP-TC10. Halo-tagged and SNAP-tagged proteins are self-labeling constructs and are commonly used for super-resolution microscopy (12). SNAP-TC10 was labeled with JF-549-SNAP and Exo70-Halo with JF-646-Halo.

TC10 overexpression results in increased mobility of Exo70 in nonpolar cells

First, we determined the impact of TC10 on Exo70 mobility in nonpolar HeLa cells. Using TIRF microscopy,

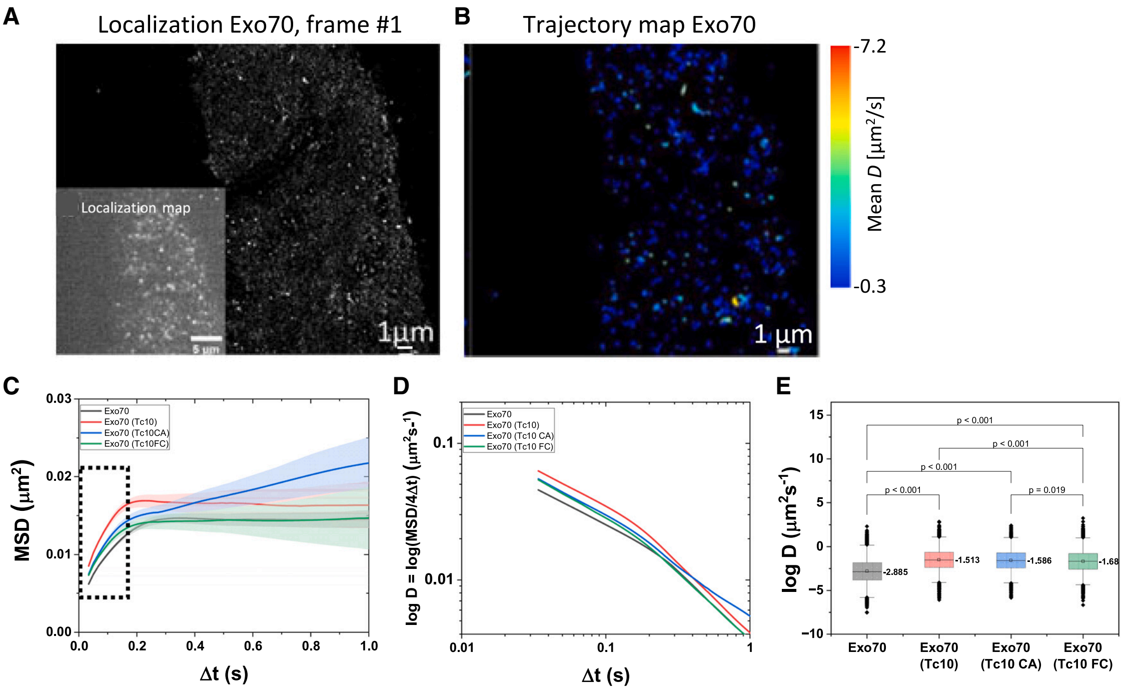

FIGURE 1 Dynamics of Exo70 in the presence of TC10 and its mutants in HeLa cells. (*A*) Localization of Exo70 in HeLa cells. One frame shows single Exo70 particles. Inset: cumulative localization map of Exo70, 5000 frames, frame rate: 17 ms, pixel size: 65 nm. (*B*) Trajectory map of Exo70 (85 s). (*C*) Ensemble time-averaged MSD curves for Exo70 (*black curve*), Exo70 with TC10 (*red curve*), Exo70 with TC10CA (*blue curve*), and Exo70 with TC10FC (*green curve*). The shaded area in the corresponding color represents the standard error of the mean. (*D*) Changes of diffusion coefficients (logarithmic) over time. (*E*) Comparison of the Exo70 diffusion in the presence of different TC10 variants. The median values of the diffusion coefficients are presented. The box limit indicates the 25th and 75th percentiles; whiskers extend to 1.5 times the interquartile range of the 25th and 75th percentiles; a straight line in the boxplot gives the median values, and this value is presented next to the box; and the significant *p*-values were estimated using the Kruskal-Wallis ANOVA test followed by post hoc analysis. $n = 5$.

we excited Exo70 exclusively at the PM. We recorded 5000 frames with a frame rate of 59 Hz, localized the molecules, and generated trajectories (Fig. 1, *A* and *B*). From these, Exo70 mobility was determined. In addition to wild-type TC10, two TC10 mutants with different activity based on the GDP/GTP state were generated using point mutations. The TC10 point mutated at Q67L mutant was permanently in the GTP binding state and thus constitutively active (TC10CA) (13). The mutant TC10F42L rapidly switches between GDP and GTP states and thus is fast cycling (TC10FC) (14). MSDs were calculated for each trajectory. We compared ensemble time-averaged MSDs for Exo70 in the presence of TC10 and its variants in the short time diffusion. As typical for particles at the PM, a longer recording shows confinement (Fig. 1 *C*). Calculation of MSD versus time on a logarithmic scale shows the deviation from normal behavior (Fig. 1 *D*). To determine the diffusion coefficient *D* ($-\log D$; $\mu m^2/s$), log MSDs were fitted with a linear function as given by

$$\log (MSD(\tau)) = \log (2dD\tau^{\alpha}) = \alpha \log \tau + \log 2d\,D,$$

where *D* is the diffusion coefficient, τ is the time, and α is the anomalous coefficient (15). The α classifies trajectories in terms of whether they exhibit normal diffusion, active transport, anomalous diffusion, or confined diffusion (16). Anomalous diffusion shows a nonlinear dependency between the MSD $\langle r^2 \rangle$ and the time (τ). An asymptotic behavior of $\langle r^2 \rangle$ at large τ indicates that the diffusive particle is restricted within a confined area. Time averaged mean square displacement extracted from trajectories of individual particles were fitted with the MSD model for the first 200 ms. R2 is a measure of the goodness of fit of a model, in this case MSD fitting model. In regression, the R2 coefficient of determination is a statistical measure of how well the regression predictions approximate the real data points. An R2 of 1 indicates that the regression predictions perfectly fit the data. These fits provided the values of *D* for individual tracks according to the models described by Qian (Fig. 1 *E*; Table S1). In HeLa cells, TC10 and TC10 mutants increased the mobility of Exo70 at the PM, whereby TC10 has the strongest effect in the short-term range. Looked at over a longer period of time, TC10CA changes the behavior of Exo70 the most: in the

presence of TC10CA, the confinement of the diffusion was practically lifted.

TC10 decreases Exo70 mobility in the axonal growth cone of cortical neurons

Next, we determined the dynamics of Exo70 in the axonal growth cone of cortical neurons. One dish at a time containing cortical neurons was transfected with Exo70-HaloTag and SNAP-TC10 (1), transfected with Exo70-HaloTag and SNAP-TC10CA (2), and co-transfected with Exo70-HaloTag and SNAP-TC10FC (3) at day in-vitro 1 DIV1. SPT was conducted at DIV2 in the growth cone, which is the site of membrane expansion. We only analyzed neurons with one axon. Single particles of Exo70 were recorded in the absence and presence of TC10, TC10CA, and TC10FC, respectively (Fig. 2 *A*). Movies of 5000 frames were recorded (58.8 Hz acquisition), and particles were localized. The superimposition of all Exo70 particles resulted in a localization map (Fig. 2 *B*). From the data, trajectories were generated. Individual trajectories were color coded based on the mean velocity of the particle. All trajectories of one movie were superimposed to generate a trajectory map (Fig. 2 *C*). We determined the MSD (Fig. 2 *D*) and plotted MSD versus time on a logarithmic scale (Fig. 2 *E*) to show the deviation from normal behavior. Exo70 mobility was significantly decreased in the presence of TC10 (Table S2). TC10CA and TC10FC had similar effects on Exo70 dynamics in the growth cones of cortical axons at DIV2 (Fig. 2 *D*). Diffusion coefficients of Exo70 were significantly lower in the presence of TC10 and active TC10 variants (Table S2). This indicates an immobilization of Exo70 due to vesicle tethering at the PM, promoted by TC10 and its active derivatives (6,7). Also, in the soma of the cortical neurons, TC10 overexpression reduced Exo70 mobility at the PM (Figs. S2 and S3). However, the radius of confinement of Exo70 differed, reflecting that Exo70 occupies different PM domains in the soma and growth cone.

Exo70 partially colocalizes with actin

The pattern of the localization and trajectory maps suggest that Exo70 may be localized along actin filaments (Fig. 2 *C*). A functional interaction of actin and Exo70 is well known for migrating and invasive cells, where the exocyst directly interacts with Arp2/3 (17). Knockdown of Exo70 has been shown to alter the actin cytoskeleton and inhibit invadopodia formation and cell migration (17,18). To test for colocalization, we stained endogenous Exo70 in hippocampal neurons by immunostaining and visualized actin by FITC-phalloidin. We found a Pearson coefficient of $\sim$0.84 (Fig. S4). When the actin skeleton in HeLa cells was destroyed with latrunculin, Exo70 mobility increased (Fig. S5), demonstrating that Exo70 diffusion is hindered due to interaction with actin, likely via Arp2/3 (17).

TC10 has no impact on Exo70 dynamics in growth cones of hippocampal neurons

To determine the effect of TC10 on Exo70 dynamics in the growth cone of hippocampal neurons, hippocampal neurons from E18 rat embryos were transfected with Exo70-HaloTag without or with SNAP-TC10 and its variants, and the Exo70 dynamics was determined as before by SPT. In the case of hippocampal neurons, the Exo70 dynamics in the growth cone was not affected by TC10 (Fig. 3; Table S3), suggesting that TC10 plays a minor role in Exo70 dynamics.

TC10 increases the fraction of Exo70 particles showing anomalous motion in cortical neurons

The logD/Δt plots of Exo70 diffusion (Figs. 1 *E*, 2 *E*, and 3 *E*) showed nonlinear behavior, indicating anomalous diffusion. Anomalous diffusion can be caused by obstacles, structural constraints of the microcompartment, or interactions that affect diffusion behavior, such as increased crowding (19). To dissect that further, we determined the α from MSD curves for all conditions (Fig. 4). α is the anomalous diffusion exponent, which typically takes a value less than 1 for subdiffusive behavior, as is common in confined motion. α were lowest for Exo70 diffusion in the cortical growth cone compared to HeLa cells and hippocampal neurons (Fig. 4 *A*). The fraction of Exo70 particles with anomalous motion ($\alpha < 1$) was highest in cortical axons (Fig. 4 *B*). Under the influence of TC10 and its mutants, this number increased in cortical neurons. TC10 had no effect on the diffusion behavior of Exo70 in hippocampal neurons, consistent with no change in D in the presence of TC10 and its functional variants (Fig. 3). In HeLa cells, the number of anomalously diffusing Exo70 particles decreased in the presence of TC10 and its mutants. Taken together, these data confirm that TC10 affects Exo70 dynamics in axonal growth cones of cortical neurons but not in hippocampal neurons.

DISCUSSION

In this study, we compared the impact of the regulatory Rho-GTPase TC10 on the dynamics of Exo70 at the PM in nonpolarized cells and in the axonal growth

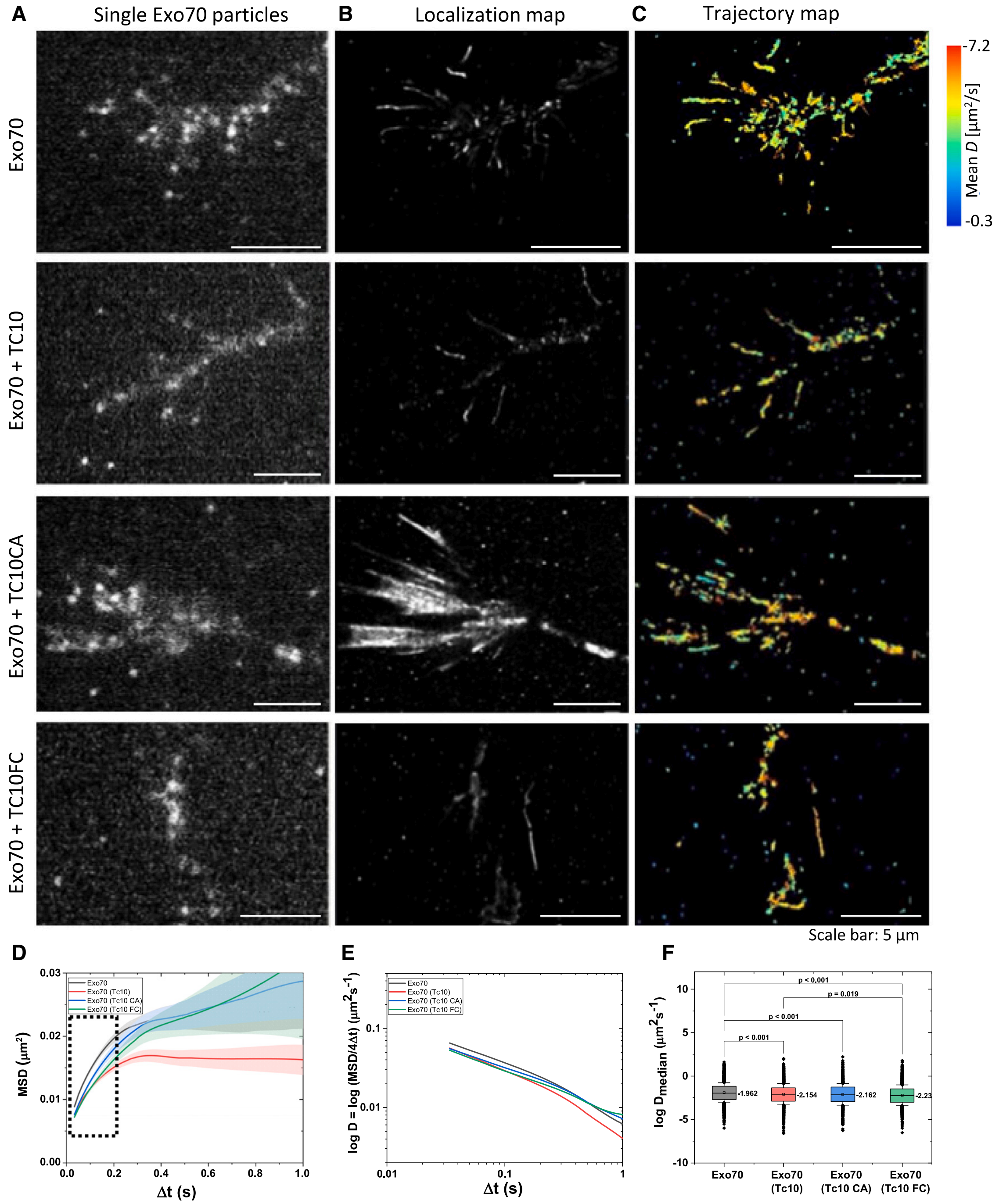

FIGURE 2 Effect of TC10 on Exo70 dynamics in the axonal growth cone of cortical neurons. (*A*) Single-particle imaging of Exo70 in the absence and presence of TC10 and TC10 variants in the growth cone; a single frame is shown. (*B*) Cumulative image of 5000 frames with localized Exo70 particles. (*C*) Trajectory map of Exo70 in the absence and presence of TC10 and TC10 variants. Each trajectory is labeled in a color related to the mean diffusion coefficient. Cumulative image of 5000 frames recoded with 58.8 Hz. (*D*) Characterization of Exo70 diffusion in the growth cone: mean-square displacements (MSDs) over time. (*E*) Change of the diffusion coefficient *D* over time ($-\log D$). (*F*) Diffusion coefficients as $-\log D$. $N = 2$.

cone of cortical and hippocampal neurons during polarization. The axonal growth cone is a zone of membrane expansion that requires the interaction of the activating Rho-GTPase TC10 with the exocyst subunit Exo70 (6). In HeLa cells, TC10 increased the diffusion of Exo70. Since our HeLa cells were not epidermal growth factor stimulated (5), the TC10 effect on Exo70 likely concerns a population that is not incorporated into the exocyst complex and has function beyond exocytosis (20). For cortical neurons, we

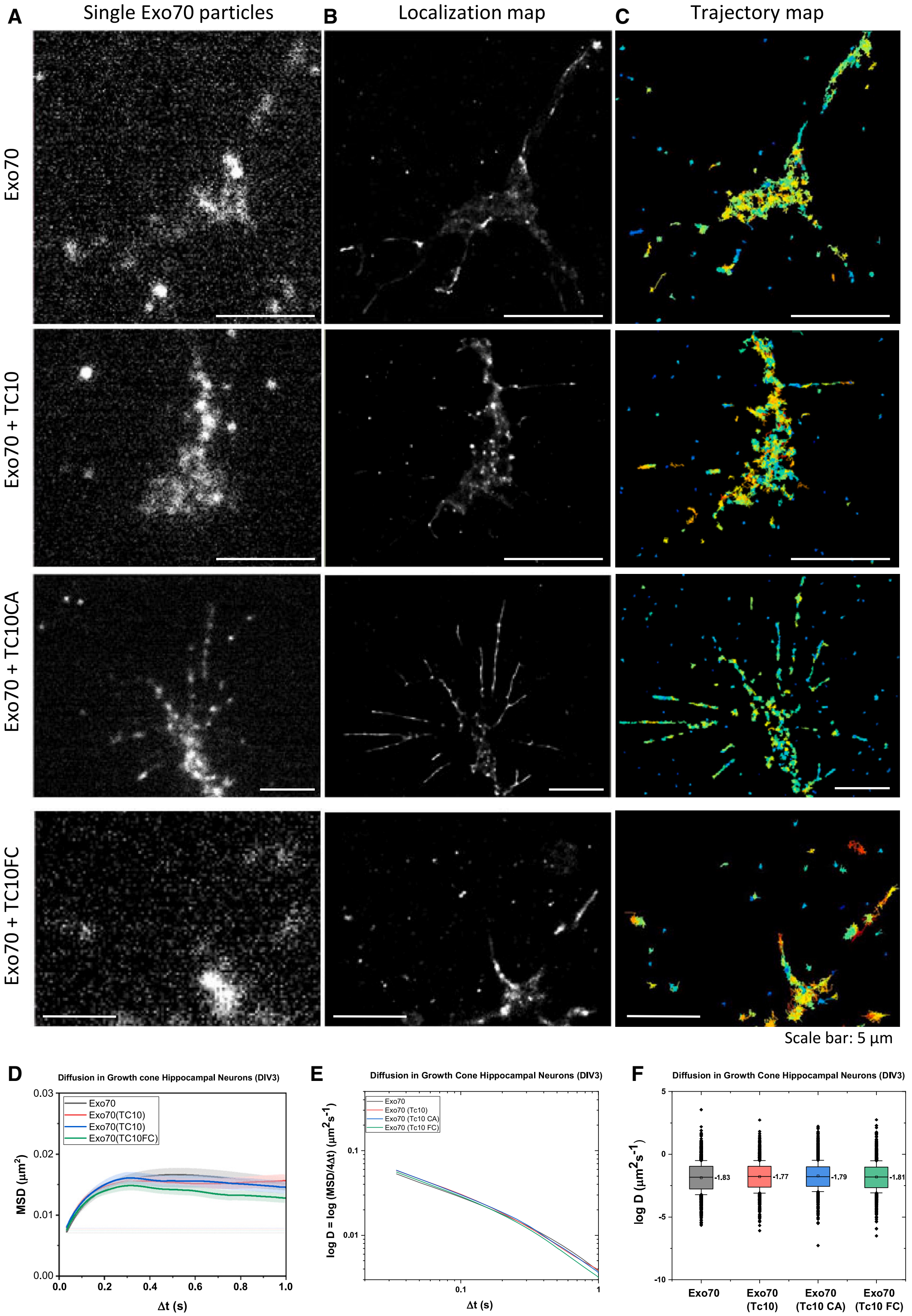

FIGURE 3 Effect of TC10 on Exo70 dynamics in the axonal growth cone of hippocampal neurons. (*A*) Single-particle imaging of Exo70 in the absence and presence of TC10 and TC10 variants in the growth cone; a single frame is shown. (*B*) Cumulative image of 5000 frames with localized Exo70 particles. (*C*) Trajectory map of Exo70 in the absence and presence of TC10 and TC10 variants. Each trajectory is labeled in a color related to the mean diffusion coefficient. Cumulative image of 5000 frames recoded with 58.8 Hz. (*D*) Characterization of Exo70 diffusion in the growth cone: mean-square displacements (MSDs) over time. (*E*) Change of the diffusion coefficient *D* over time ($-\log D$). (*F*) Diffusion coefficients as $-\log D$. $N = 2$. Scale bars: 5 μm (*A*–*C*).

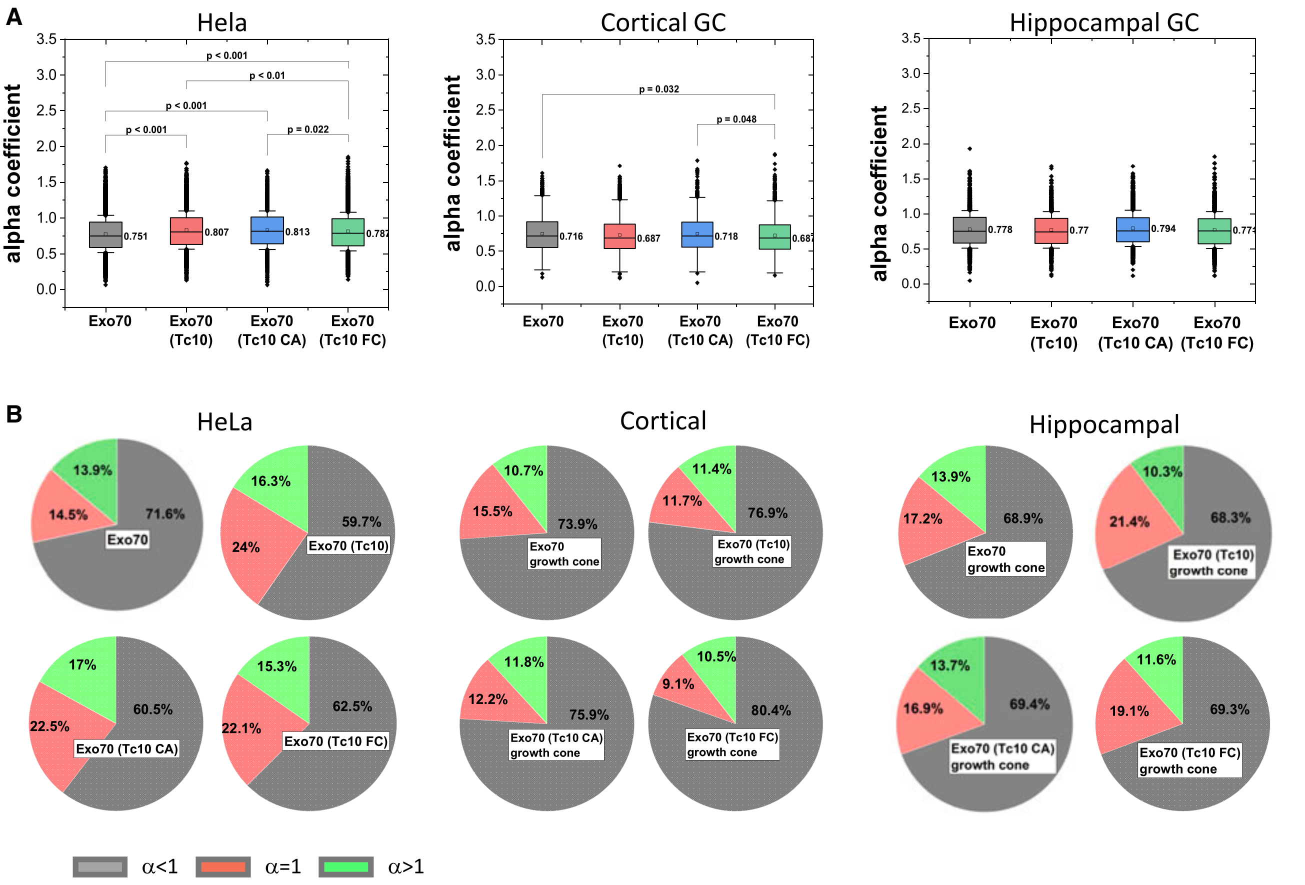

FIGURE 4 Anomalous diffusion of Exo70 at the plasma membrane. (*A*) Mean alpha coefficients (α) for anamolus diffusion of Exo70 in HeLa cells and the growth cone of cortical and hippocampal neurons in the absence and presence of TC10 and its variants. Boxplots present the mean values of the α, and the box limit indicates the 25th and 75th percentiles; whiskers extend to 1.5 times the interquartile range of the 25th and 75th percentiles; a square in the boxplot gives the mean values, and this value is presented next to the box; and the significant *p*-values are estimated using one ANOVA test followed by Tukey's test analysis. Replicates: $N = 5$ (HeLa), $N = 2$ (cortical neurons), and $N = 3$ (hippocampal neurons). (*B*) Dissection of motion behavior. The pie chart shows the respective population of Exo70 following anomalous or confined motion (*gray part*, $\alpha < 1$), Brownian motion (*red part*, $\alpha = 1$), and directed motion (*green portion*, $\alpha > 1$).

found that TC10 decreased the diffusion of Exo70 in the soma and the growth zone. Moreover, the fraction of Exo70 that displayed anomalous diffusion ($\alpha < 1$) was increased. The reduced mobility of Exo70 linked to TC10 expression can be explained by enhanced Exo70/exocyst vesicle tethering. In this scenario, GTP-TC10 interacts with Exo70 to promote vesicle binding. The GTP-TC10 variant TC10-Q67L, which is constitutively active (TC10CA), had the same effect as TC10 on Exo70 mobility. However, the fast-cycling variant TC10/34L increased the fraction of Exo70 displaying confined motion in accordance with the promotion of vesicle fusion (7). The confinement radius of Exo70 was larger in the growth cone than in the soma or HeLa cells. This is likely determined by the interaction of Exo70 with the cytoskeleton, which forms extended patterns in the growth cone (21, 22). In contrast to the growth cone of cortical neurons, overexpression of TC10 had no effect on Exo70 dynamics in the growth cone of hippocampal neurons. Although a previous study reported TC10 control of Exo70 in hippocampal neurons (6), it was recently demonstrated that axonal growth is also regulated by Cdc42b (8). Apparently, the integrated action of both Rho-family GTPases is specifically crucial in axonal outgrowth of hippocampal neurons.

DATA AND CODE AVAILABILITY

All data supporting the conclusions of this article are included within the manuscript, and raw data and movies are available upon reasonable request to the corresponding author.

ACKNOWLEDGMENTS

The authors were members of the CIM. K.B. and H.G. were funded by GRF grant #386797833 (SFB1348). H.G. was supported by GRC grant #386797833. We are thankful to Priyadarshini Ravindran and

Andreas Püschel for providing transfected neurons and the Exo70 and TC10 mutants. We also would like to thank Frank Schmelter for sample preparation and Nico Marx for data analysis.

AUTHOR CONTRIBUTIONS

H.G. and K.B. designed experiments. H.G. conducted the experiments. K.B. and H.G. analyzed data. K.B. and H.G. wrote the manuscript.

DECLARATION OF INTERESTS

The authors declare no competing interests.

SUPPORTING MATERIAL

Supplemental information can be found online at https://doi.org/10.1016/j.bpr.2024.100186.

REFERENCES

1. Barnes, A. P., and F. Polleux. 2009. Establishment of axon-dendrite polarity in developing neurons. *Annu. Rev. Neurosci.* 32:347–381.
2. Dotti, C. G., C. A. Sullivan, and G. A. Banker. 1988. The establishment of polarity by hippocampal neurons in culture. *J. Neurosci.* 8:1454–1468.
3. Rakic, P., L. J. Stensas, …, R. L. Sidman. 1974. Computer-aided three-dimensional reconstruction and quantitative analysis of cells from serial electron microscopic montages of foetal monkey brain. *Nature.* 250:31–34.
4. Affenzeller, M. J., A. Darehshouri, …, U. Lütz-Meindl. 2009. Salt stress-induced cell death in the unicellular green alga Micrasterias denticulata. *J. Exp. Bot.* 60:939–954.
5. Kawase, K., T. Nakamura, …, M. Matsuda. 2006. GTP hydrolysis by the Rho family GTPase TC10 promotes exocytic vesicle fusion. *Dev. Cell.* 11:411–421.
6. Dupraz, S., D. Grassi, …, S. Quiroga. 2009. The TC10-Exo70 complex is essential for membrane expansion and axonal specification in developing neurons. *J. Neurosci.* 29:13292–13301.
7. Fujita, A., S. Koinuma, …, T. Nakamura. 2013. GTP hydrolysis of TC10 promotes neurite outgrowth through exocytic fusion of Rab11- and L1-containing vesicles by releasing exocyst component Exo70. *PLoS One.* 8:e79689.
8. Ravindran, P., and A. W. Puschel. 2023. An isoform-specific function of Cdc42 in regulating mammalian Exo70 during axon formation. *Life Sci. Alliance.* 6:e202201722.
9. Tinevez, J.-Y., N. Perry, K. W. Eliceiri…, 2017. TrackMate: An open and extensible platform for single-particle tracking. *Methods.* 115:80–90. https://doi.org/10.1016/j.ymeth.2016.09.016.
10. Tarantino, N., J.-Y. Tinevez, E. Laplantine…, 2014. TNF and IL-1 exhibit distinct ubiquitin requirements for inducing NEMO-IKK supramolecular structures. *J. Cell Biol.* 204:231–245. https://doi.org/10.1083/jcb.201307172.
11. Qian, H., M. P. Sheetz, and E. L. Elson. 1991. Single particle tracking. Analysis of diffusion and flow in two-dimensional systems. *Biophys. J.* 60:910–921. https://doi.org/10.1016/S0006-3495(91)82125-7.
12. Liss, V., B. Barlag, …, M. Hensel. 2015. Self-labelling enzymes as universal tags for fluorescence microscopy, super-resolution microscopy and electron microscopy. *Sci. Rep.* 5:17740.
13. Inoue, M., L. Chang, …, A. R. Saltiel. 2003. The exocyst complex is required for targeting of Glut4 to the plasma membrane by insulin. *Nature.* 422:629–633.
14. Lopez Tobon, A., M. Suresh, …, K. D. Fischer. 2018. The guanine nucleotide exchange factor Arhgef7/betaPix promotes axon formation upstream of TC10. *Sci. Rep.* 8:8811.
15. Saxton, M. J. 2008. A biological interpretation of transient anomalous subdiffusion. II. Reaction kinetics. *Biophys. J.* 94:760–771.
16. Ruthardt, N., D. C. Lamb, and C. Bräuchle. 2011. Single-particle tracking as a quantitative microscopy-based approach to unravel cell entry mechanisms of viruses and pharmaceutical nanoparticles. *Mol. Ther.* 19:1199–1211.
17. Liu, J., P. Yue, …, W. Guo. 2009. The role of the exocyst in matrix metalloproteinase secretion and actin dynamics during tumor cell invadopodia formation. *Mol. Biol. Cell.* 20:3763–3771.
18. Ma, W., Y. Wang, …, M. Yin. 2016. The role of Exo70 in vascular smooth muscle cell migration. *Cell. Mol. Biol. Lett.* 21:20.
19. Guigas, G., and M. Weiss. 2008. Influence of hydrophobic mismatching on membrane protein diffusion. *Biophys. J.* 95:L25–L27.
20. Zhu, Y., B. Wu, and W. Guo. 2019. The role of Exo70 in exocytosis and beyond. *Small GTPases.* 10:331–335.
21. Fritzsche, M., D. Li, …, C. Eggeling. 2017. Self-organizing actin patterns shape membrane architecture but not cell mechanics. *Nat. Commun.* 8:14347.
22. Nozumi, M., F. Nakatsu, …, M. Igarashi. 2017. Coordinated Movement of Vesicles and Actin Bundles during Nerve Growth Revealed by Superresolution Microscopy. *Cell Rep.* 18:2203–2216.

Biophysical Reports, Volume *4*

Supplemental information

TC10 differently controls the dynamics of Exo70 in growth cones of cortical and hippocampal neurons

Hiteshika Gosain and Karin B. Busch

Supporting Material

TC10 differently controls the dynamics of Exo70 in growth cones of cortical and hippocampal neurons

Running title: Exo70 dynamics controlled by TC10

Hiteshika Gosain[1], Karin B. Busch[1]*

[1]Institute of Integrative Cell Biology and Physiology, Department of Biology, University of Muenster, 48149 Münster, North-Rhine-Westphalia, Germany

*Corresponding author

Karin Busch, e-mail: buschkar@uni-muenster.de

Supplementary Methods

Co-Localization of Exo70 and actin

Hippocampal neurons were a gift from A. Püschel. Exo70 was immune-stained with anti-Exo70 (rb) (Proteintech (12014-1-AP) using a dilution of 1:50, the 2nd antibody was anti-rb-Alexa555 (Invitrogen A21147) used in a dilution of 1:500. Actin was stained with Alexa488-Phalloidin (1 µM, Invitrogen A12379). Exo70/Alexa555 was excited with 551 nm, Alexa488-Phalloidin with 488 nm. Imaging was performed with an Elyra7-TIRF microscope. Two aligned Hamamatsu ORCA-Fusion BT sCMOS cameras were used to image fluorescent Exo70 and actin.

Supplementary Figures

Figure S 1: Colocalization of Exo70 and TC10 in HeLa cells. (A) Endogenous TC10 (green) and (b) endogenous Exo70 (red), immune-staining. (c) Merge of Exo70 and TC10. TC10 and Exo70 are found both at the plasma membrane and as puncta. (d, e, f) Zoomed in regions of figures (a, b, c) (white boxes), size 100x100 pixels. Colocalization of Exo70 and TC10 are shown as yellow spots in (c) and (f). Primary/secondary antibody are Exo70/AF555, TC10/AF488. (g) The threshold for both the green (TC10) and red (Exo70) channels was adjusted, and colocalization was performed in Imaris™ (Bitplane) in an area of 100x100 pixels. Colocalization is indicated by white spots. (h) Scatter plot to determine the Pearson's coefficient. (i) Calculated colocalization (%) averaged over 30 images (100x100 px). Scale bars: 10 µm (a,b,c), 1 µm (d,e,f).

Figure S 2: **Effect of TC10 on Exo70 dynamics in the soma of cortical neurons**. (A) Single particle imaging of Exo70 in the absence and presence of TC10 and TC10 variants in the soma, a single frame is shown. (B) Cumulative image of 5000 frames with localized Exo70 particles. (C) Trajectory map of Exo70 in the absence and presence of TC10 and TC10 variants. Each trajectory is labelled in a colour related to the mean diffusion coefficient. Cumulative image of 5000 frames recoded with 58.8 Hz. (D) Characterization of Exo70 diffusion in the soma: mean square displacements (MSD) over time. (E) Change of the diffusion coefficient *D* over time (-log D). (F) Diffusion coefficients as -log *D*. The box limit indicates the 25th and 75th percentiles; whiskers extend to 1.5 times the interquartile range of the 25th and 75th percentiles; a straight line in the box plot gives the median values and this value is

presented next to the box; the significant p values were estimated using the Kruskal-Wallis ANOVA test followed by post-hoc analysis. N=2.

Figure S 3: Confinement and anomalous diffusion of Exo70 at the plasma membrane in HeLa cells and cortical neurons. (A) Means square displacements. (B) Dissection of motion behavior. The pie chart shows the respective population of Exo70 following anomalous or confined motion (grey part, $\alpha<1$), Brownian motion (red part, $\alpha=1$) and directed motion (green portion, $\alpha>1$).

Figure S 4: **Partial co-localization of Exo70 and actin in hippocampal neurites.** (A) Immunolabeled endogenous Exo70 and FITC-Phalloidin stained actin show co-localization in hippocampal neurons. (B) Several regions of interest (rois) were analyzed for colocalization using the Colocalization ImageJ plugin. The Pearson's colocalization factor was calculated (graph). Each dot represents data from a single roi.

Figure S 5: Exo70 mobility in HeLa cells is influenced by actin. Destruction of the actin skeleton in HeLa cells by latrunculin and its effect on Exo70-HaloTag mobility shown as means square displacement (MSD) over time. Framed area indicates short-term movement.

Figure S 1

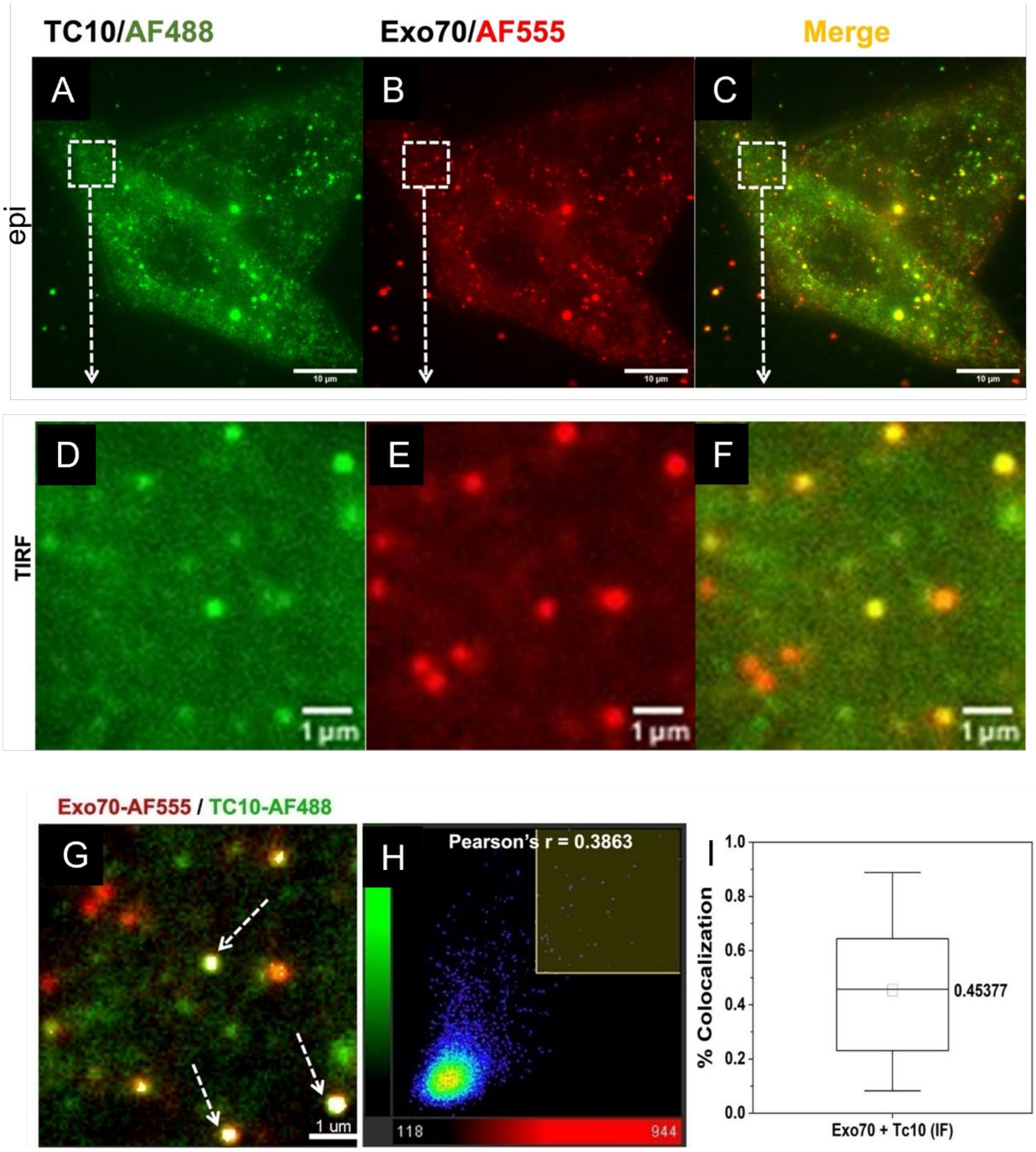
TC10/AF488
Exo70/AF555
Merge
A
B
C
epi
10 µm
D
E
F
TIRF
1 µm
Exo70-AF555 / TC10-AF488
G
1 um
H
Pearson's r = 0.3863
118
944
I
% Colocalization
1.0
0.8
0.6
0.4
0.2
0.0
0.45377
Exo70 + Tc10 (IF)

Figure S 2

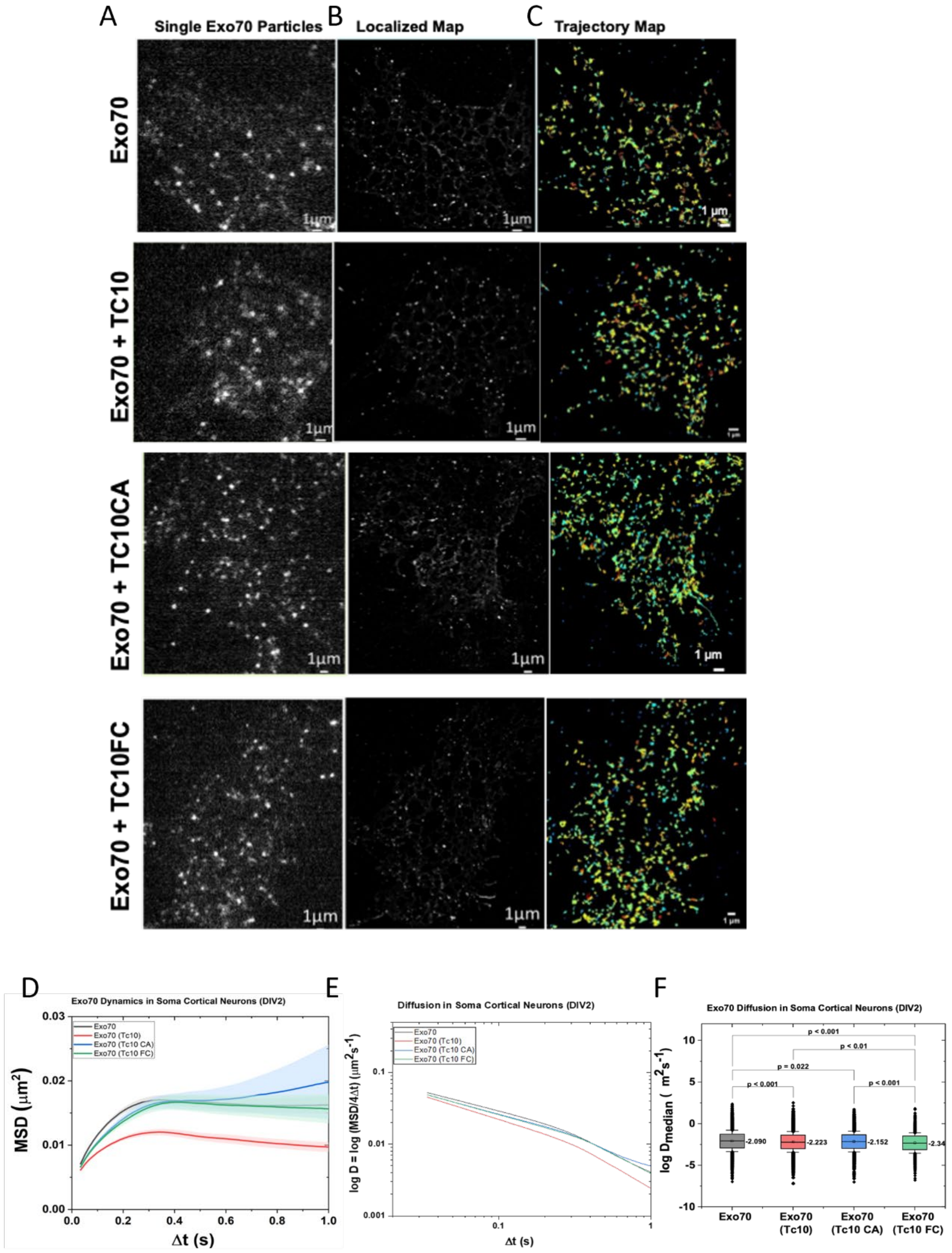
A
Single Exo70 Particles
B
Localized Map
C
Trajectory Map
Exo70
Exo70 + TC10
Exo70 + TC10CA
Exo70 + TC10FC
1μm
1 μm
D
Exo70 Dynamics in Soma Cortical Neurons (DIV2)
Exo70
Exo70 (Tc10)
Exo70 (Tc10 CA)
Exo70 (Tc10 FC)
MSD (μm²)
Δt (s)
E
Diffusion in Soma Cortical Neurons (DIV2)
log D = log (MSD/4Δt) (μm²s⁻¹)
Δt (s)
F
Exo70 Diffusion in Soma Cortical Neurons (DIV2)
p < 0.001
p < 0.01
p = 0.022
p < 0.001
p < 0.001
log Dmedian (m²s⁻¹)
-2.090
-2.223
-2.152
-2.34
Exo70
Exo70 (Tc10)
Exo70 (Tc10 CA)
Exo70 (Tc10 FC)

Figure S 3

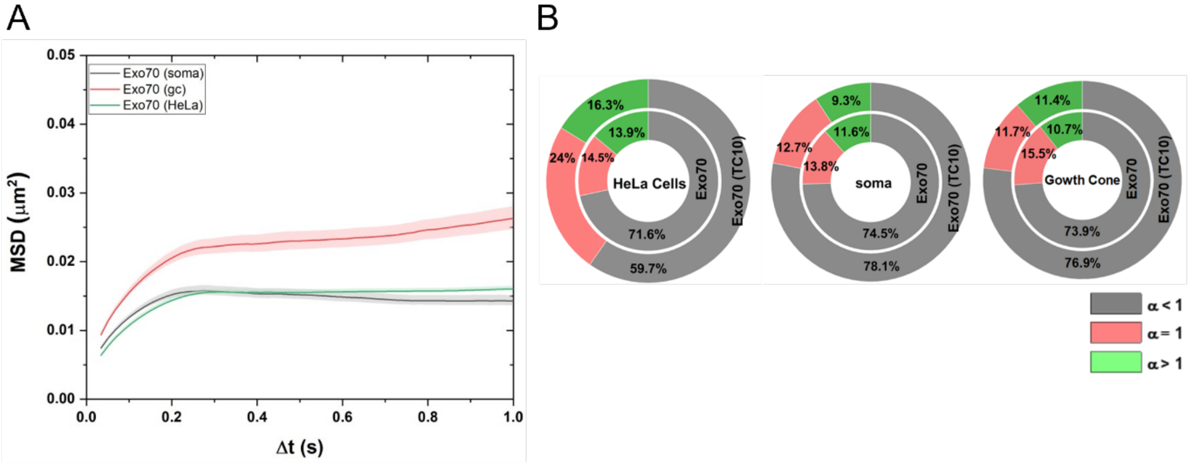
A
B
0.05
0.04
0.03
0.02
0.01
0.00
MSD (μm²)
0.0
0.2
0.4
0.6
0.8
1.0
Δt (s)
Exo70 (soma)
Exo70 (gc)
Exo70 (HeLa)
HeLa Cells
Exo70
Exo70 (TC10)
16.3%
13.9%
24%
14.5%
71.6%
59.7%
soma
9.3%
11.6%
12.7%
13.8%
74.5%
78.1%
Gowth Cone
11.4%
10.7%
11.7%
15.5%
73.9%
76.9%
α < 1
α = 1
α > 1

Figure S 4

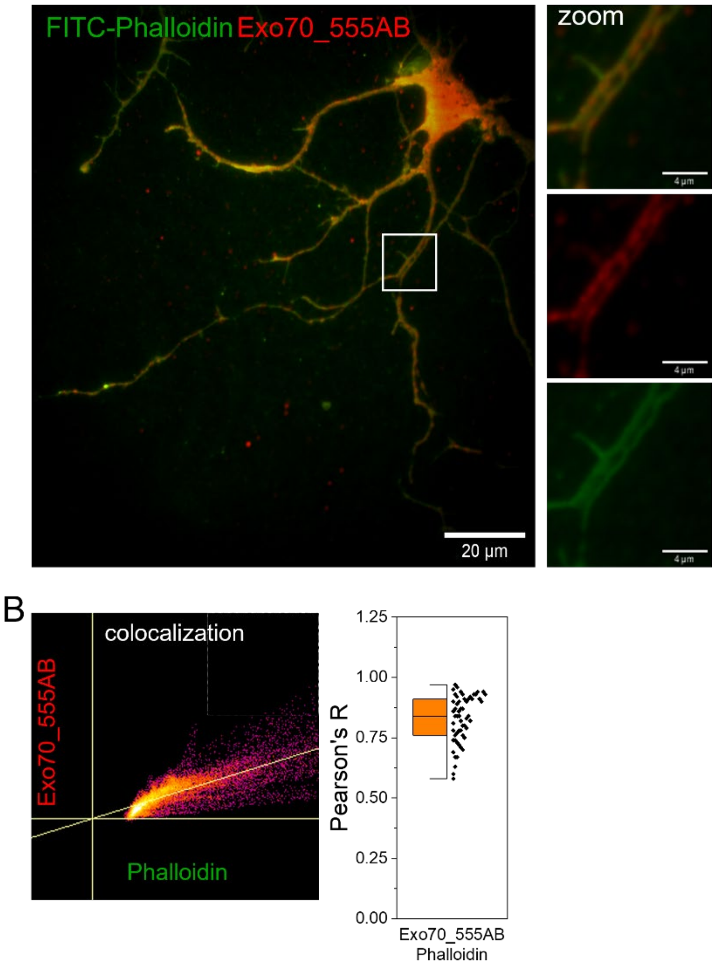
FITC-Phalloidin Exo70_555AB
zoom
20 µm
4 µm
4 µm
4 µm
B
colocalization
Exo70_555AB
Phalloidin
Pearson's R
1.25
1.00
0.75
0.50
0.25
0.00
Exo70_555AB
Phalloidin

Figure S 5

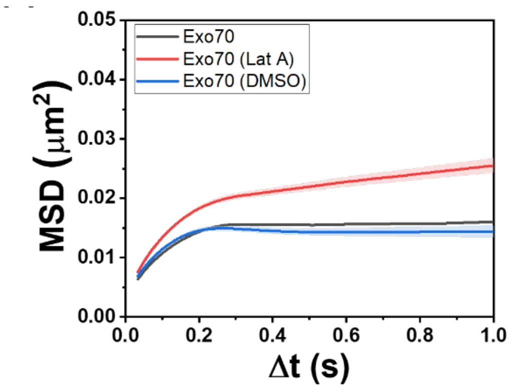

Exo70
Exo70 (Lat A)
Exo70 (DMSO)
MSD (μm²)
0.05
0.04
0.03
0.02
0.01
0.00
0.0
0.2
0.4
0.6
0.8
1.0
Δt (s)

Supplementary Tables

Table 1: Effect of TC10 on the diffusion of Exo70 in nonpolar HeLa cells, N=3.

HeLa cells	Total # tracks	$\mathcal{D}_{median}$±s.e.m. (μm²s⁻¹)	α_{mean} ± s.e.m.	Number of cells
Exo70	22724	0.011 ± 0.001	0.776 ± 0.003	20
Exo70 (TC10)	11644	0.031 ± 0.002	0.832 ± 0.005	20
Exo70 (TC10CA)	9249	0.026 ± 0.003	0.830 ± 0.005	20
Exo70 (TC10FC)	16188	0.021 ± 0.002	0.811 ± 0.004	20

Table 2: Effect of TC10 on the diffusion of Exo70 in the axonal growth cone of cortical neurons, N=2.

Growth Cone Cortical Neurons (DIV2)	Total tracks	$\mathcal{D}_{median}$±s.e.m. ($\mu m^2 s^{-1}$)	α_{mean} ± s.e.m.	Number of cells
**Exo70**	6938	0.011 ± 0.002	0.749 ± 0.006	13
**Exo70 (TC10)**	9108	0.007 ± 0.003	0.731 ± 0.006	13
**Exo70 (TC10CA)**	4402	0.007 ± 0.005	0.745 ± 0.007	12
**Exo70 (TC10FC)**	4949	0.006 ± 0.003	0.724 ± 0.007	12

Table 3: Effect of TC10 on the diffusion of Exo70 in the axonal growth cone of hippocampal neurons. N=2.

Growth Cone Hippocampal Neurons (DIV3)	Total tracks	$\mathcal{D}_{median}$±s.e.m. ($\mu m^2 s^{-1}$)	α_{mean} ± s.e.m.	Number of cells
**Exo70**	5544	0.015 ± 0.001	0.778 ± 0.007	9
**Exo70 (TC10)**	3407	0.017 ± 0.002	0.770 ± 0.009	8
**Exo70 (TC10CA)**	5229	0.016 ± 0.003	0.794 ± 0.008	8
**Exo70 (TC10FC)**	4552	0.016 ± 0.003	0.771 ± 0.008	8
